# Depressive and anxiety symptoms amid COVID-19 pandemic among healthcare workers in a low-resource setting: a systematic review and meta-analysis from Ethiopia

**DOI:** 10.3389/fpsyt.2024.1342002

**Published:** 2024-10-22

**Authors:** Tilahun Kassew, Mamaru Melkam, Woredaw Minichil, Mesele Wondie, Dawed Ali

**Affiliations:** Department of Psychiatry, College of Medicine and Health Sciences, University of Gondar, Gondar, Ethiopia

**Keywords:** anxiety, depression, COVID-19 pandemic, meta-analysis, systematic review, Ethiopia

## Abstract

**Background:**

Coronavirus disease 2019 (COVID-19) outbreak is one of the public health problems that pose a serious mental health concern due to its high morbidity and mortality rate. The healthcare workers are at risk of developing mental health symptoms like depression and anxiety because they are the first point of contact in the diagnosis, treatment, and care of patients with COVID-19. This study aimed to systematically review the prevalence and the associated factors of depression and anxiety disorders among healthcare workers amid COVID-19 pandemic in Ethiopia.

**Method:**

A systematic review and meta-analysis study was conducted. Different primary studies that assessed the depressive and anxiety disorders during amid COVID-19 pandemic in the Ethiopian healthcare workers were extracted by Microsoft Excel and exported to STATA version 11 for further analysis. Random-effects model meta-analysis was used to the estimate pooled effect size and the effect of each study with their 95% confidence interval. Funnel plot analysis and Egger regression tests were conducted to detect the presence of publication bias. Subgroup analysis and sensitivity analysis were conducted.

**Results:**

Thirteen studies with 5,174 participants were included in this systematic review and meta-analysis study. The pooled prevalence of depression and anxiety disorders was 40.39% (95% CI: 28.54, 52.24) and 44.93% (95% CI: 31.39, 58.46), respectively. Being a woman, being married, working in the frontline, and having high perceived susceptibility were significantly associated with depression among the Ethiopian healthcare workers. Similarly, being a woman, being older in age, working in the frontline, and having high perceived susceptibility were the factors associated with anxiety disorder among the Ethiopian healthcare workers during the COVID-19 pandemic.

**Conclusion:**

The prevalence of depression and anxiety disorders in the Ethiopian healthcare workers was high. The timely detection and appropriate management of mental health problems is essential for the quality of healthcare services, and proactive support methods for the female, married, and older-age healthcare professionals could result in these outcomes.

**Systematic Review Registration:**

https://www.crd.york.ac.uk/PROSPERO/, identifier CRD42022299074.

## Introduction

Coronavirus disease 2019 (COVID-19) is a family of ribonucleic acid (RNA) viruses that results in infections for humans with respiratory syndrome, causing unprecedented numbers of deaths and substantial psychological distress to the global community (1, 2). The diseases rapidly propagated, and, in a realm of less than a month, it rapidly disseminated in other parts of the world; hence, the detected cases were reported in nine countries (3), and, then, it spread and nocked the door of the majority of countries. Since then, a staggering number of new cases are pouring into the case box every single minute, and death toll is climbing overwhelmingly high with a growth factor above 1 indicating an exponential increase (4). Ethiopia has faced the pandemic and reported the case of COVID-19 in March 2020 (5). The shock wave of information regarding the pandemic in the country is more than ever since the first case was confirmed.

The outbreak of the COVID-19 pandemic results in a huge mental health concern such as depression, anxiety, adjustment disorder, panic attacks, and insomnia because of the fear of getting sick and/or dying (6–8). The psychological fear reaction results from the infection and death of higher numbers of people by COVID-19 infection in the general public (9). The healthcare workers (HCWs) are at risk of developing mental health symptoms like depression and anxiety because they are the first point of contact in the diagnosis, treatment, and care of patients with COVID-19 (10, 11). Recent literature studies reported that the disease outbreak causes a higher level of mental health impacts particularly depression and anxiety symptoms among HCWs compared to those in the general population (12–14). In regard to the types of mental health impact, depression, anxiety, stress, and insomnia were ranging from 10.6% to 58%, 11.1% to 100%, 5.2% to 100%, and 28.75% to 34%, respectively (10, 15–17). Factors, such as gender, age, educational level, work experience, psychological/medical comorbidity, perceived social support, perceived susceptibility, working environment, and personal/family exposure, were the factors associated with the negative mental state of HCWs due to COVID-19 (10, 18–20).

Higher levels of depressive and anxiety symptoms are also associated with the perceived susceptibility to the disease and perceived severity of disease (21, 22) and loneliness (23, 24). World Health Organization reports that depression, anxiety, panic disorder, suicide, and sleep disturbances increased because of the burden of psychological distress in the world (25). Furthermore, it reports that COVID‐19 pandemic–related long‐lasting depression and anxiety problems are becoming a serious public health concern (26). These mental health problems can prevent the HCWs from fulfilling their duties and reduce the quality of healthcare services to patients (27, 28).

In Ethiopia, there are inconsistent primary studies on mental health problems and its correlates amid COVID-19 outbreak on HCWs, which need comprehensive evidence for decision-making, and, yet, there is no systematic review and meta-analysis. Studying the mental health impacts of COVID-19 gives vital information on its prevalence of depression and anxiety symptoms and enables us to discover HCWs dynamics of strong resilience. This information helps to the planning and provision of preventive strategies and effective treatment modalities to strengthen its positive outcomes and health policy of the country to address the HCWs’ mental health outcomes of pandemic. Thus, the aim of this systematic review and meta-analysis was to assess the prevalence of depression and anxiety symptoms and its potential correlates on HCWs during the COVID-19 response in Ethiopia.

## Methods

We conducted a systematic review to identify the prevalence of depressive and anxiety symptoms and its correlates in low-resource settings using the Preferred Reporting Items for Systematic Review and Meta-Analysis (PRISMA) guidelines (29). The checklist of meta-analysis of observational study in epidemiology was also followed. The project’s protocol was prepared, registered, and published on Prospero with the registration number CRD42022299074.

### Search strategy

We rigorously searched the peer-reviewed literature studies that were found from PubMed, Medline, ScienceDirect, and Google Scholar. The electronic databases were searched for all the published articles until 15 March 2022. In addition to these searching databases, the reference lists of relevant articles were manually searched. PubMed database was searched using the search term ((Mental health symptoms [MeSH Terms]) OR (negative mental outcomes) OR (Depression) OR (depressive symptoms) OR (anxiety) OR (anxiety symptoms) OR (Anxiety disorders) OR (panic attack) OR (stress)) AND ((healthcare workers) [MeSH Terms]) OR (healthcare professionals)) AND (corona-virus disease 2019) [MeSH Terms]) OR (COVID-19) OR COVID-19 pandemic)) AND Ethiopia. Again, Medline and ScienceDirect databases were searched using similar search terms as applied in PubMed. Google Scholar was also searched for gray literature and published paper in unindexed journals.

### Selection of the studies

**Figure 1:**
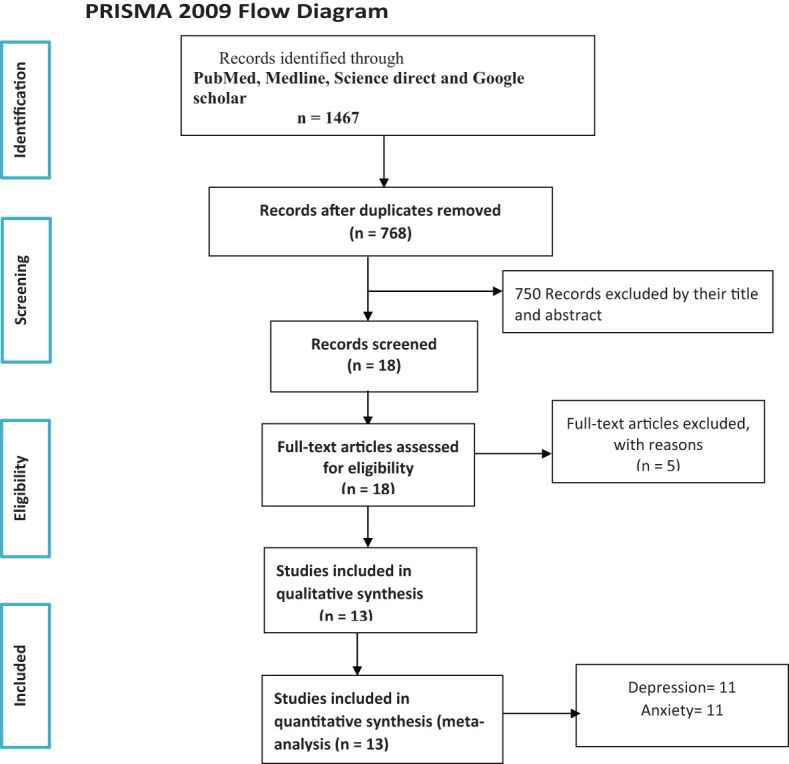
Flowchart of the systematic research and study selection process.

Two blinded researchers (TK and MM) searched for the published articles. All citations (N = 13) identified in the search process using their titles and abstracts were imported into Endnote X7.8 reference management software. This software was also used to eliminate any duplicates. Wherever there was a disagreement of relevancy of the included studies during the review, the reviewers have discussed together to reach consensus. A flow diagram (**Figure 1**) was used to show the included studies.

### Definition of the variables

In this review, depression was a variable that was assessed using the validated standardized screening tools on the Ethiopian HCWs during COVID-19 pandemic. The tools used for measurement of depression in the included studies were the Depression, Anxiety and Stress Scale–21 (DASS-21) scale (30, 31) and Patient Health Questionnaire 9 (PHQ-9) (32). In addition, anxiety was also an outcome variable that was measured using DASS-21 (30, 31) and the seven-item Generalized Anxiety Disorder–7 (GAD-7) (33) in the included studies.

### Eligibility criteria

The studies fulfilling the following criteria were included for the review: cross-sectional studies that assess one or more mental health symptoms (depression and anxiety) due to the current COVID-19 pandemic; the articles published in English language; and the studies interested on data of the Ethiopian HCWs. The articles with the unclear outcome of interest, case reports, reviews, and qualitative studies were excluded. The Joanna Briggs Institute study quality checker was used to evaluate the quality of the paper included (34). A quality appraisal criterion was used to check studies conducted by using cross-sectional research and prevalence data. The final reviewed paper was five or above out of a total of nine rating scales. This quality appraisal score was assessed by two investigators (TK and MM), and disagreements were solved by discussion.

### Data extraction and synthesis

The required data of the included articles were extracted by using a structured and customized data abstraction sheet and stored in a Microsoft Excel spreadsheet. The data were extracted by two independent researchers (TK and DA) and checked for accuracy by the two independent reviewers and by MW and WM as a mediator. The extracted data include the author’s name, year of publication, study area, study design, aims, sample size, methods of data collection, primary outcomes (depression and anxiety), and their correlates. Magnitude of depression and anxiety symptoms amid COVID-19 and the associated factors with 95% confidence intervals (CIs) were also extracted. A narrative synthesis was employed to describe the studies design, implementation, and findings.

### Data processing and analysis

The extracted data were entered into Microsoft Excel and then exported to STATA version 14 for further analysis. Random-effects model meta-analysis was used to estimate the pooled effect size and the effect of each study with their 95% CI. A forest plot was used to determine the pooled effect size and magnitude of each recruited study with 95% CI to indicate a graphic summary of the data. The index of heterogeneity (I^2^ statistics) was used to measure the degree of heterogeneity among the included studies (35). The potential sources of heterogeneity were identified through subgroup analysis by (region, study setting, and measurement tool), and a sensitivity analysis was also conducted to determine the potential source of heterogeneity. Funnel plot analysis and Egger-weighted regression tests were conducted to detect the presence of publication bias. P-value < 0.05 in Egger’s test was considered evidence of statistically significant publication bias (36, 37).

## Results

### Search results

The articles were searched from PubMed, Medline, ScienceDirect, and Google Scholar. From the searching databases, 1,467 articles were found initially. A total of 699 articles were removed because of duplication; later, 768 articles were left. After seeing their titles and abstracts, 750 articles were removed. Therefore, only 18 articles underwent a full-text review. Finally, we included 13 articles that fulfilled the inclusion criteria to conduct this systematic review and meta-analysis, whereas five studies were excluded from the review as they did not satisfy the eligibility criteria (**Figure 1**).

### Characteristics of the included studies

After screening, 13 studies (13, 14, 38–48) with a total of 5,174 participants were included in the analysis. All of the studies were cross-sectional and reported on the prevalence and/or associated factors of depression and anxiety disorders on the Ethiopian HCWs during the COVID-19 pandemic. From the included studies, 11 studies that assessed depression and/or associated factors were included in this systematic review and meta-analysis (**Table 2**). Similarly, 11 studies that assessed the anxiety disorders and/or the associated factors were included in this systematic review and meta-analysis as indicated in **Table 1**. Nine of the studies assessed the outcome variable with a self-administered questionnaire, two studies with a face-to-face interview, and two studies using an online survey method of data collection. PHQ-9 (13, 39, 41, 43, 46) and DASS-21 (14, 40, 42, 44, 45, 47) were the frequently used tools for the screening of depression. On the other hand, GAD-7 (13, 38, 39, 43, 46, 48) and DASS-21 (14, 40, 44, 45, 47) were the two most common assessment instruments for the assessment of anxiety.

**Table 1:** Characteristics of the included studies on depression and anxiety disorders amid COVID-19 pandemic on the Ethiopian healthcare workers.

Author, year	Region	Study setting	Sample size	Measurement tool	Data collection method	Depression and anxiety prevalence (%)	Quality score
Yadeta et al. (2021) (41)	Oromia	Primary healthcare settings	265	PHQ-9	Interview	D = 66.4	8
Wayessa et al. (2021) (42)	Oromia	Primary healthcare settings	275	DASS-21	Interview	D = 21.5	6
Jemal et al. (2020) (14)	Oromia	Primary healthcare settings	816	DASS-21	Self-administered	D = 60.3 A = 78	9
Habtamu et al. (2021) (13)	Addis Ababa	Hospitals	238	PHQ-9 and GAD-7	Self-administered	D = 27.3 A = 31.1	9
Mulatu et al. (2021) (43)	Addis Ababa	Hospitals	420	PHQ-9 and GAD-7	Self-administered	D = 20.2 A = 21.9	8
Mekonen et al. (2020) (44)	Amhara	Hospitals	302	DASS-21	Self-administered	D = 55.3 A = 69.6	9
Asnakew et al. (2021) (45)	Amhara	Primary healthcare settings	419	DASS-21	Self-administered	D = 58.2 A = 64.7	9
Ayalew et al. (2021) (40)	SNNPs	Hospitals	387	DASS-21	Self-administered	D = 50.1 A = 55	7
GebreEyesus et al. (2021) (46)	SNNPs	Hospitals	322	PHQ-9 and GAD-7	Self-administered	D = 25.8 A = 36	8
Hajure et al. (2021) (47)	Oromia	Primary healthcare settings	127	DASS-21	Self-administered	D = 43.3 A = 51.2	6
Deriba et al. (2021) (39)	Oromia	Primary healthcare settings	417	PHQ-9 and GAD-7	Online survey	D = 16.3 A = 30.7	8
Teshome et al. (2020) (38)	SNNPs	Primary healthcare settings	798	GAD-7	Self-administered	A = 29.3	7
Dagne et al. (2020) (48)	Amhara	Hospitals	388	GAD-7	Online survey	A = 26.8	6

A, anxiety; D, depression; DASS-21, Depression, Anxiety and Stress Scale–21; GAD-7, Generalized Anxiety Disorder–7; PHQ-9, Patient Health Questionnaire–9.

**Table 2:** Sensitivity analysis on prevalence of depression and anxiety in HCWs during COVID-19 pandemic in Ethiopia.

Study omitted, year	Estimate (95% CI)	
	Depression	Anxiety
Yadeta et al. (2021) (41)	37.79 (25.82, 49.78)	----
Wayessa et al. (2021) (42)	42.29 (29.72, 54.86)	----
Habtamu et al. (2021) (13)	41.70 (28.95, 54.45)	46.30 (31.87, 60.74)
Mulatu et al. (2021) (43)	42.43 (30.01, 54.85)	47.24 (33.35, 61.14)
Mekonen et al. (2020) (44)	38.91 (26.33, 51.48)	42.46 (28.27, 56.65)
Asnakew et al. (2021) (45)	38.61 (26.18, 51.03)	42.95 (28.49, 57.40)
Ayalew et al. (2021) (40)	39.42 (26.59, 52.26)	43.92 (29.19, 58.65)
GebreEyesus et al. (2021) (46)	41.86 (29.06, 54.66)	45.82 (31.19, 60.45)
Hajure et al. (2021) (47)	40.11 (27.50, 52.72)	44.32 (29.94, 58.69)
Jemal et al. (2020) (14)	38.37 (26.67, 50.08)	41.56 (30.97, 52.14)
Deriba et al. (2021) (39)	42.83 (31.11, 54.54)	46.36 (31.78, 60.93)
Dagne et al. (2020) (48)	----	46.75 (32.39, 61.11)
Teshome et al. (2020) (38)	----	46.50 (31.91, 61.10)
Combined	40.39 (28.54, 52.24)	44.93 (31.39, 58.46)

A summary of the characteristics of the included studies based on the author’s name and year of publication, region, study setting, sample size, measurement tool, methods of data collection, and main findings is provided in **Table 1**.

### The prevalence of depression

**Figure 2:**
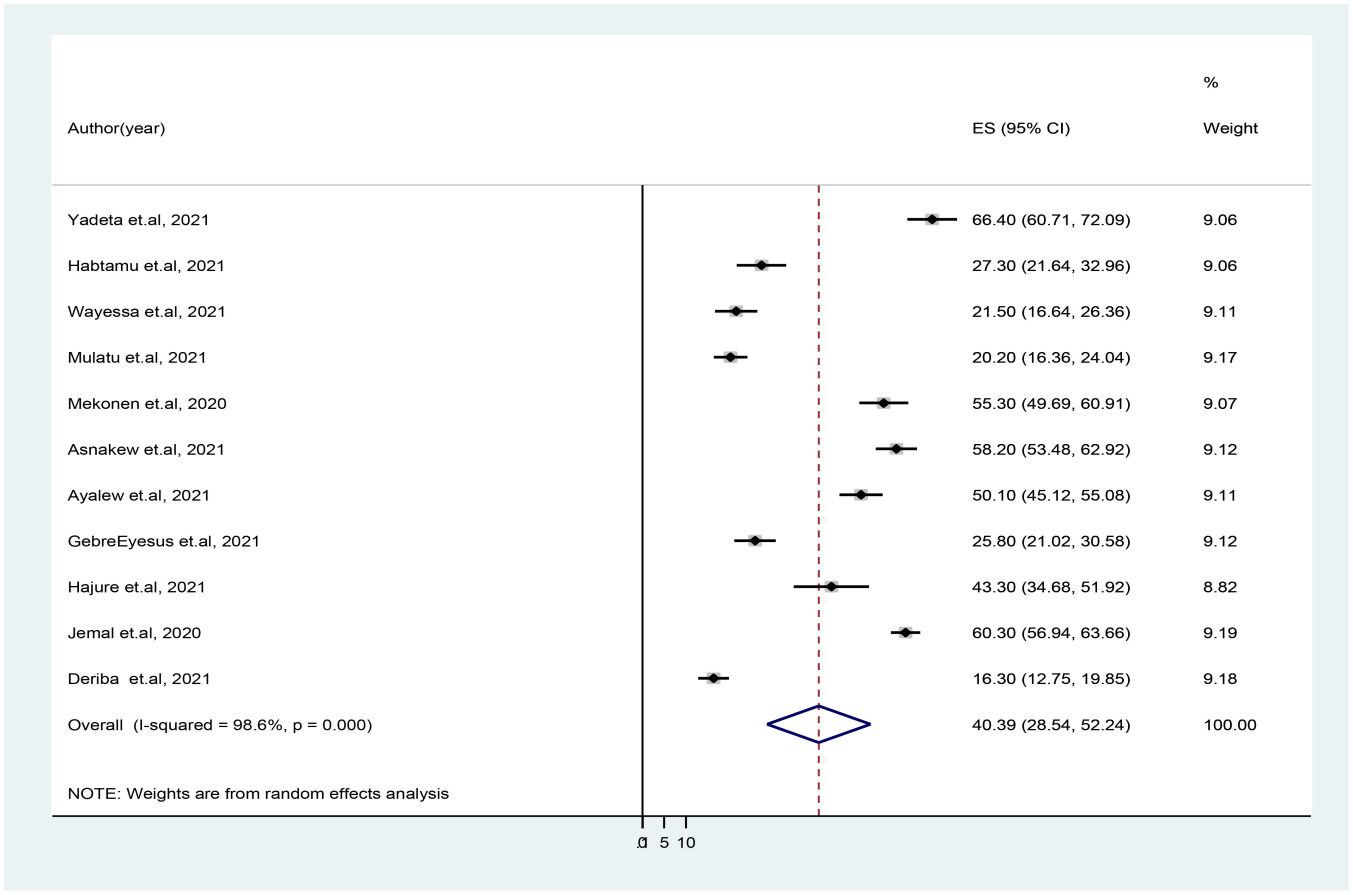
Forest plot showing the pooled prevalence of depression in healthcare workers during COVID-19 pandemic in Ethiopia. Note: The midpoint and the length of each segment indicate prevalence and a 95% confidence interval (CI), whereas the diamond shape shows the combined prevalence of all studies.

In the 11 included studies, the pooled prevalence of depression in HCWs during COVID-19 pandemic was 40.39% (95% CI: 28.54, 52.24) as indicated in **Figure 4**. There was a significant level of heterogeneity among the included studies (*I^2^* = 9 8.6%, p ≤ 0.001) (**Figure 2**). A sensitivity analysis was performed by omitting each study individually to see whether each one affected the average prevalence because of the significant level of heterogeneity of the meta-analysis. This illustrates that the omission of any one of the studies from this systematic review and meta-analysis does not change the overall pooled prevalence of depression, as all point values are within the overall 95% CI (**Table 2**).

**Figure 3:**
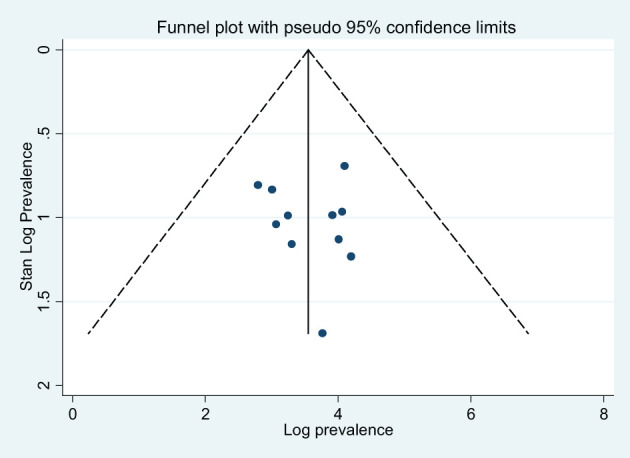
Funnel plots for publication bias of the studies that were included in the prevalence of depression in Ethiopia.

A subgroup analysis by region, measurement tool, and study setting was performed to identify the source of heterogeneity. Accordingly, five studies were conducted in Oromia region, and the pooled prevalence of depression in these studies was 41.53% (95% CI: 19.57, 63.49, *I* ^2^ = 99.1%). The prevalence of depression was higher in studies conducted at the primary healthcare settings (44.31%) than that in studies conducted at referral and/or specialized hospitals (35.69%). Furthermore, the average prevalence of depression of HCWs amid COVID-19 was 48.18%, as it is measured by using DASS-21 measurement tool. The level of heterogeneity among studies included in each subgroup analysis was high (p < 0.05), except that among the studies conducted in Amhara region as illustrated in **Table 3**. The funnel plot test is symmetric (**Figure 3**), and the Eggers test for publication bias was insignificant [*B* = 5.91 (95% CI: −19.74, 31.56), *SE* = 11.34, and *P* = 0.62], illustrating that there was no evidence of publication bias for the prevalence of depression.

**Figure 4:**
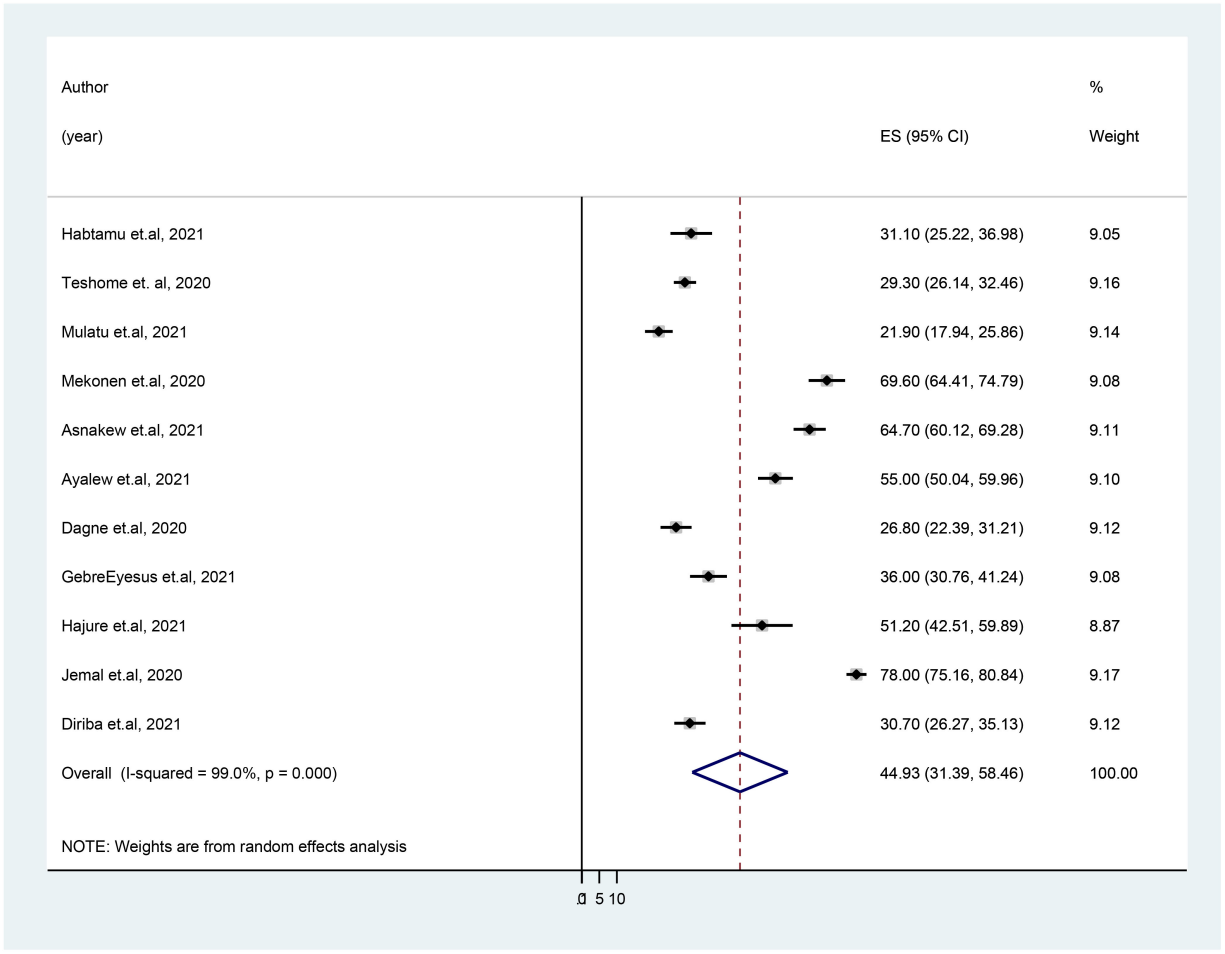
Forest plot showing the pooled prevalence of anxiety disorder in healthcare workers during COVID-19 pandemic in Ethiopia.

**Table 3:** The subgroup analysis on the prevalence of depressive and anxiety disorders in HCWs during COVID-19 in Ethiopia.

Variables	Subgroup	Number of studies	Sample size	Prevalence (95% CI)	I^2^ (%)	P- value
Subgroup analysis for the prevalence of depression						
Region	Oromia	5	1,900	41.53 (19.57, 63.49	99.1	≤0.001
	Amhara	2	721	57 (53.38, 60.61)	0.0	0.438
	SNNPs	2	709	37.94 (14.13, 61.75)	97.9	<0.001
	Addis Ababa	2	658	23.43 (16.50, 30.36)	75.8	0.042
Study setting	Primary healthcare	6	2,319	44.31 (25.49, 63.14)	99	<0.001
	Hospitals	5	1,669	35.69 (21.81, 49.56)		<0.001
Measurement tool	PHQ-9	5	1,662	31.11 (15.69, 46.53)	98.3	<0.001
	DASS-21	6	2,326	48.18 (35.89, 60.47)	97.3	<0.001
Subgroup analysis for the prevalence of anxiety						
Region	Oromia	3	1,360	53.34 (19.27, 87.41)	99.4	<0.001
	Amhara	3	1,109	53.68 (26.52, 80.84)	99.0	<0.001
	SNNPs	3	1,507	40.03 (24.66, 55.41)	97.3	<0.001
	Addis Ababa	2	658	26.23 (17.23, 35.23)	84.6	0.011
Study setting	Primary healthcare	5	2,577	50.79 (27.97, 73.60)	99.4	<0.001
	Hospitals -	6	2,057	40.04 (25.16, 54.92)	98.2	<0.001
Measurement tool	GAD-7	6	2,583	29.08 (25.42, 32.75)	76.3	0.001
	DASS-21	5	2,051	64.04 (54.13, 73.95)	95.5	<0.001

SNNPs, Southern Nation Nationalities and Peoples; DASS-21, Depression, Anxiety and Stress Scale–21; GAD-7, Generalized Anxiety Disorder–7; PHQ-9, Patient Health Questionnaire–9.

### The prevalence of anxiety disorder

**Figure 4** shows that, among Ethiopian healthcare professionals during the COVID-19 pandemic, the pooled prevalence of anxiety disorder was 44.93% (95% CI: 31.39, 58.46) across the 11 studies that were included. This meta-analysis revealed heterogeneity (I^2^ = 98.6%, p = 0.001); as a result, a sensitivity analysis was carried out by omitting each study independently to determine whether doing so had an impact on the average prevalence. This illustrates that the omission of any one of the studies from this systematic review and meta-analysis does not influence the overall pooled prevalence of anxiety disorder, as all point values are within the overall 95% CI (**Table 2**).

**Figure 5:**
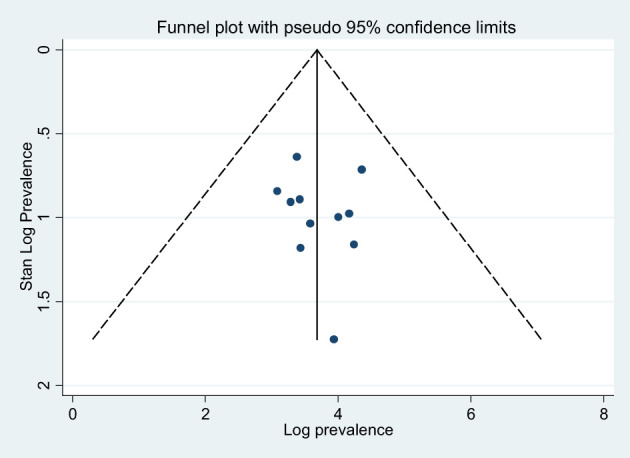
Funnel plots for publication bias of the studies that were included in the prevalence of disorder in Ethiopia.

A subgroup analysis by region, study setting, and measurement tool was performed to identify the source of heterogeneity. Accordingly, the pooled prevalence of anxiety disorder in the Amhara region studies was 53.68% (95% CI: 26.52, 80.84), whereas, in Addis Ababa, it was 26.23% (95% CI: 17.23, 35.23). The prevalence of anxiety disorder was higher in studies conducted at the primary health settings (health centers and primary hospitals) (50.79%) than that in studies conducted at referral and specialized hospitals (40.04%). Furthermore, the average prevalence of anxiety disorder in HCWs amid COVID-19 was 64.04% as measured by using the DASS-21 measurement tool. The level of heterogeneity among studies included in each subgroup analysis was high (p < 0.01) as illustrated in **Table 3**. The funnel plot test is symmetric (**Figure 5**), and the Eggers test for publication bias was insignificant [*B* = −7.19 (95% CI: −34.01, 19.62), *SE* = 11.83, and *P* = 0.56], indicating that there was no evidence of publication bias for the prevalence of anxiety symptoms.

### Factors associated with depressive and anxiety disorders

In this meta-analysis study, five factors (being a woman, being married, working in the frontline, having high perceived susceptibility, and having poor social support) and four factors (being a woman, being older in age, working in the frontline, and having high perceived susceptibility) were extracted to evaluate the determinant factors with depression and anxiety, respectively. Female participants were 2.5 and 2.11 times more likely to have depressive and anxiety disorders than male participants [adjusted OR (AOR) = 2.50 (95% CI: 1.89–3.31)] and [AOR = 2.11 (95% CI: 1.69–2.64)], respectively. Participants who worked at the frontline during COVID-19 were 2.22 and 2.84 times more likely to have depressive and anxiety symptoms than participants who work other than frontline healthcare activities [AOR = 2.22 (95% CI: 1.57–3.15) and AOR = 2.84 (95% CI: 2.19–3.69)], respectively. The odds of depressive and anxiety disorders, respectively, were 3.25 and 2.34 times higher among HCWs with high perceived susceptibility to COVID-19 infection than those with low perceived susceptibility. Furthermore, the odds of depressive disorder was three times higher among married participants than unmarried HCWs [AOR = 3.02 (95% CI: 2.19–4.16)], and older participants [AOR = 3.87 (95% CI: 1.72–8.71)] were significantly more likely to have anxiety disorder amid COVID-19 pandemic than young HCWs (**Table 4**).

**Table 4:** Odds ratio of factors associated with depressive and anxiety disorders in Ethiopian healthcare workers amid COVID-19.

Variables for depression	AOR (95% CI)	I^2^	P-value
**Being a woman**	2.5 (1.89–3.31)	18.2%	0.29
**Frontline work**	2.22 (1.57–3.15)	0.0%	0.99
**High susceptibility**	3.25 (2.01–5.24)	0.0%	0.57
**Being married**	3.02 (2.19–4.16)	38.6%	0.16
**Poor social support**	1.09 (0.74–1.59)	0.0%	0.98
Variables for anxiety			
**Being a woman**	2.11 (1.69–2.64)	0.0%	0.43
**Frontline work**	2.84 (2.19–3.69)	0.0%	0.58
**High susceptibility**	2.34 (1.62–3.38)	0.0%	0.70
**Older age**	3.87 (1.72–8.71)	0.0%	0.35

AOR, adjusted odds ratio; CI, confidence interval; I^2^, heterogeneity.

## Discussion

The current review is the first quantitative epidemiological review of depression and anxiety disorders among HCWs during the COVID-19 pandemic in Ethiopia. We systematically identified 13 cross-sectional studies and quantitatively synthesized the pooled prevalence of depressive and anxiety symptoms. The overall prevalence of depression and anxiety was 40.39% and 44.93%, respectively. This study confirmed the presence of a high prevalence of depression and anxiety problems among HCWs in Ethiopia. It is common to observe higher mental health problems in healthcare providers due to the widespread occurrence of COVID-19 pandemic and increased number of cases and deaths and more vulnerable to becoming infected or even transmitting the disease to their family members (49–53). This study’s value for the prevalence of anxiety and depressive disorders was in line with other reviews and meta-analyses. For instance, according to a review by Hossain et al. (2021), during the COVID-19 pandemic, the prevalence of depression and anxiety disorders among HCWs was higher in Pakistan (depression, 41.6%; and anxiety, 50.4%) and Bangladesh (depression, 48.2%; and anxiety, 43.6%) (54). Similarly, the global estimated prevalence of depression and anxiety disorders among HCWs was predicted to be 37% and 40%, respectively, according to the review and meta-analysis by Saragih et al. (2021) (55). The prevalence of depression and anxiety among nurses was also reported by Al Maqbali et al. (2021) to be 35% and 37%, respectively (56).

The estimated prevalence of depression and anxiety disorders in this study was higher than that in the earlier review and meta-analysis conducted in high resources settings. For example, in a review and meta-analysis done by Pappa et al. (2020) on healthcare professionals, the estimated prevalence of depression and anxiety was 22.8% and 23.2%, respectively (57). The findings in this study are also higher than those in the recently published meta-analysis findings, with the estimated prevalence of depression of 21.7% (58), 24% (59), 26% (60), and 24.83% (61), in which most of the participants were from high-resource settings. Furthermore, the estimated prevalence of anxiety in this study also is higher than that in the earlier published meta-analysis findings with the estimated prevalence ranging from 22.1% to 30% (58, 61–63).

The possible reason for these different prevalences might be due to the dissimilarities in infrastructural facilities of healthcare systems and the level of attention given to the mental wellbeing of HCWs by policymakers and health authorities. This variation may be due to the difference in study period. The earlier review and meat-analyses were done in the early period of the COVID-19 pandemic during which there were limited studies in the low-resource settings (53). A variety of rating scales used the studies are the other possible reason for the source of variability among study findings. It is evident by the results of the subgroup analysis that the prevalence of depression and anxiety disorders in studies measured via DASS-21 (48.18% and 64.04%, respectively) was higher than those measured using PHQ-9 and GAD-7 scales. The variation in sensitivity and specificity of the rating scales to screen out depression and anxiety could be responsible for this. On the other hand, an increasing trend of infected and hospitalized patients leads to a heavier workload, threatening the mental health of the Ethiopian HCWs. Furthermore, the shortage of personal protective equipment frequently reported in the low-resource settings like Ethiopia can increase the mental health impacts and lead to stress reactions of HCWs during the COVID-19 pandemic (64, 65). This indicates the importance of empowering healthcare professionals to successfully deal with the emotions rising from such difficult circumstances.

The HCWs in Addis Ababa had a lower prevalence of both conditions (depression, 23.43%; and anxiety, 26.23%) compared to those studies held in other regions of the country in this review and meta-analysis. For instance, the HCWs in Amhara region had a higher prevalence of both conditions (depression, 57%; and anxiety, 53.68%) compared to those in Addis Ababa (depression, 23.43%; and anxiety, 26.23%). The possible explanation for this epidemiological variation of depression and anxiety disorders across regions may be attributed to the variation in personal protective equipment, workload related to increased infected and hospitalized cases, and concept of the illness (66, 67). There were limited infrastructural facilities of healthcare system in other regions than that in Addis Ababa during the earlier period of the pandemic. Moreover, the average prevalence of depression and anxiety of HCWs was higher (44.31% and 50.79%, respectively) in the studies held in the primary healthcare settings than those done in hospitals.

Our review suggested that being a woman is the determinant factor for depression and anxiety disorders among HCWs during COVID-19. This finding is in line with other systematic review and meta-analysis studies (68–70). In another study by Han et al. (2020) on HCWs, the estimated prevalence of depression in women was higher than that in men (71). A possible reason might be the greater number of female workers employed globally in lower status roles within healthcare systems, which put them in greater risk of exposure to patients with COVID-19 and bring negative outcomes on mental wellbeing (60). Women are more burdened with different tasks of giving care in the office and at home than men, which brings mental health impact related to the COVID pandemic (70). It is also the fact that women had negative feeling and emotion and that the effect of hormonal difference could contribute a great role for the adverse effects of mental wellbeing (69, 72).

Being older in age was the potential risk factor for anxiety disorders among HCWs during COVID-19. Previous studies also affirmed that older-aged HCWs had the greatest anxiety disorders due to considerable changes in physical health and their responsibilities of taking care of family members and organizing their work affairs (73, 74). The other associated factor with depression in HCWs at the time of the COVID pandemic is being married. This finding is consistent with other studies in China (75, 76). The possible explanation could be the fear of contaminating and loss their loved ones due to the pandemic and separation from the family members (77, 78).

Furthermore, frontline work and high perceived susceptibility to COVID-19 infection were significant risk factors for depression and anxiety disorders among health workers. Because they are the initial point of contact in the diagnosis, treatment, and care of patients with COVID-19, HCWs are at risk for acquiring depression and anxiety (10, 11). The frontline HCW may be more susceptible to depression and anxiety disorders due to exposure to unpredictable daily caseloads, the burden of making decisions, many deaths, bans on family visits, and ongoing changes to clinical guidelines (62). This might be due to the lack of personal protective equipment, the fear of being infected or infecting family members, having chronic medical condition, contact with infected person, and feeling of helplessness that can render the work environment dangerous, leading to a feeling of insecurity and vulnerability to the infection that could have a mental health impact on HCWs (79–81). This demonstrated the necessity of providing healthcare personnel with proper personal protection equipment and psychosocial support to improve their mental wellbeing.

### Limitations

This review and meta-analysis has some limitations. First, the current research did not include mental disorders other than depression and anxiety, such as distress, trauma, and sleep disorders, which limited our findings. Second, the studies included in this review were cross-sectional studies that could not show the causal relationship of the potential risk factors with depression and anxiety disorders. Furthermore, using the broad phrase “healthcare worker” prevents us from drawing conclusions about particular occupations like that of a doctor or nurse.

## Conclusion

Our study revealed that the prevalence of depression and anxiety disorders in Ethiopian HCWs was high. Being a woman, being married, being older in age, working in the frontline, and having high perceived susceptibility were the factors associated with depressive and anxiety disorders amid COVID-19 pandemic in HCWs. Findings also provided health policymaker’s evidence-based information to improve the mental health of HCWs through earlier detection of high-risk groups based on their demographic and working characteristics, as well as the provision of emotional support programs. The timely detection and appropriate management of mental health problems is essential for the quality of healthcare services, and proactive support methods for the female, married, and older-age healthcare professionals could result in these outcomes.

## Data Availability

The original contributions presented in the study are included in the article/**Supplementary Material**. Further inquiries can be directed to the corresponding author.
