# Supplementary material for: Depressive and anxiety symptoms amid COVID-19 pandemic among healthcare workers in a low-resource setting: a systematic review and meta-analysis from Ethiopia
Source: Front Psychiatry. 2024 Oct 22;15:1342002. doi: 10.3389/fpsyt.2024.1342002 (PMC11536703; doi:10.3389/fpsyt.2024.1342002)
Supplement: Supplementary file 1 [file Table1.docx]

Table_S1 Quality assessment of “Depression and anxiety symptoms amid COVID-19 outbreak among healthcare workers in Ethiopia” included studies in this meta-analysis and systematic review

| Author, year of  Publication | Q1 | Q2 | Q3 | Q4 | Q5 | Q6 | Q7 | Q8 | Q9 | Total score (9) |
| --- | --- | --- | --- | --- | --- | --- | --- | --- | --- | --- |
| Yadeta et.al, 2021 | Y | NA | Y | Y | Y | Y | Y | Y | Y | 8 |
| Wayessa et.al, 2021 | Y | Y | NA | Y | Y | NA | NA | Y | Y | 6 |
| Jemal et.al, 2020 | Y | Y | Y | Y | Y | Y | Y | Y | Y | 9 |
| Habtamu et.al, 2021 | Y | Y | Y | Y | Y | Y | Y | Y | Y | 9 |
| Mulatu et.al, 2021 | Y | Y | Y | Y | Y | NA | Y | Y | Y | 8 |
| Mekonen et.al, 2020 | Y | Y | Y | Y | Y | Y | Y | Y | Y | 9 |
| Asnakew et.al, 2021 | Y | Y | Y | Y | Y | Y | Y | Y | Y | 9 |
| Ayalew et.al, 2021 | Y | Y | Y | Y | Y | NA | NA | Y | Y | 7 |
| GebreEyesus et.al, 2021 | Y | Y | Y | Y | NR | Y | Y | Y | Y | 8 |
| Hajure et.al, 2021 | Y | NA | NA | Y | Y | NA | Y | Y | Y | 6 |
| Deriba et.al, 2021 | Y | Y | Y | NR | Y | Y | Y | Y | Y | 8 |
| Teshome et. al, 2020 | Y | Y | Y | NR | Y | Y | Y | Y | Y | 7 |
| Dagne et.al, 2020 | Y | NR | Y | NA | NR | Y | Y | Y | Y | 6 |

**Key:** **Y**= Yes; **NR**= Not reported, **NA**=Not appropriate

**Question codes:**

1. Was the sample frame appropriate to address the target population?

2. Were study participants sampled in an appropriate way?

3. Was the sample size adequate?

4. Were the study subjects and the setting described in detail?

5. Was the data analysis conducted with sufficient coverage of the identified sample?

6. Were valid methods used for the identification of the condition?

7. Was the condition measured in a standard, reliable way for all participants?

8. Was there appropriate statistical analysis?

9. was the response rate adequate, and if not, was the low response rate managed appropriately?
